# Post traumatic stress disorder symptoms and stress burden among caregivers of patients with severe mental illness

**DOI:** 10.1192/j.eurpsy.2021.353

**Published:** 2021-08-13

**Authors:** A. Rady, T. Mouloukheya, E. Ramadan

**Affiliations:** Psychiatry, Alexandria University School of Medicine, Alexandria, Egypt

**Keywords:** ptsd, stress burden, Mental illness, care giver

## Abstract

**Introduction:**

Care givers of patients with severe mental disorders have been shown to be under heavy stress burden that reflect itself through various heterogenous psychiatric symptoms that may mimic PTSD with associated negative impact on interpersonal relations and work performance

**Objectives:**

to assess the prevalence of PTSD symptoms among care givers of patients with severe mental illness

**Methods:**

70 patients care givers of sevely mentally ill patients compred to control 70 care giver of patients with chronic debilitating medical illness were recruited from outpatient of the university hospital outpatient facilities, random selection. Severe mental illness was defined by Global assessment of function GAF score above 50 and duration exceeding 2 years. Both groups were subject to Zarit burden interview to assess stress burden and post traumatic stress diagnostic scale PDS to assess PTSD symptomats

**Results:**

43% of care givers of severly mentally patients showed moderate to severe burden on the Zarit scale compared to only 10% among care givers of medically ill patients, this difference was statistically significant (p<0,001). Among care givers of severly mental patients showed moderate to severe score on post traumatic stress diagnostic scale compared to 0% among those taking care of medically ill patients. this difference was statistically significant (p<0,001)

**Conclusions:**

Stress burden among care givers of patients with severe mental illness is high and may manifest symptoms of post traumatic disorder. This highlight the importance of particular psychological support and assessment among care givers of patients with sever mental illness

**Disclosure:**

No significant relationships.

